# Engineering of Recombinant Sheep Pox Viruses Expressing Foreign Antigens

**DOI:** 10.3390/microorganisms9051005

**Published:** 2021-05-07

**Authors:** Olga Chervyakova, Elmira Tailakova, Nurlan Kozhabergenov, Sandugash Sadikaliyeva, Kulyaisan Sultankulova, Kunsulu Zakarya, Rinat A. Maksyutov, Vitaliy Strochkov, Nurlan Sandybayev

**Affiliations:** 1 Research Institute for Biological Safety Problems, RK ME&S–Science Committee, Gvardeiskiy 080409, Kazakhstan; tailakova_86@mail.ru (E.T.); nurlanks@gmail.com (N.K.); sadikalieva86@mail.ru (S.S.); sultankul70@mail.ru (K.S.); rkm_kz@mail.ru (K.Z.); vstrochkov@gmail.com (V.S.); nurlan.s@mail.ru (N.S.); 2 State Research Center of Virology and Biotechnology “Vector”, Koltsovo, 630559 Novosibirsk Region, Russia; maksyutov_ra@vector.nsc.ru

**Keywords:** SPPV, vaccine vector, integration plasmids, thymidine kinase, ribonucleotide reductase

## Abstract

Capripoxviruses with a host range limited to ruminants have the great potential to be used as vaccine vectors. The aim of this work was to evaluate attenuated sheep pox virus (SPPV) vaccine strain NISKHI as a vector expressing several genes. Open reading frames SPPV020 (ribonucleotide kinase) and SPPV066 (thymidine kinase) were selected as sites for the insertion of foreign genes. Two integration plasmids with expression cassette were designed and constructed. Recombinant SPPVs expressing an enhanced green fluorescent protein (EGFP) (rSPPV(RRΔ)EGFP and rSPPV(TKΔ)EGFP), Foot-and-mouth disease virus capsid protein (VP1), and *Brucella* spp. outer membrane protein 25 (OMP25) (rSPPV(RRΔ)VP1A-(TKΔ)OMP25) were generated under the transient dominant selection method. The insertion of foreign genes into the SPPV020 and SPPV066 open reading frames did not influence the replication of the recombinant viruses in the cells. Successful foreign gene expression in vitro was assessed by luminescent microscopy (EGFP) and Western blot (VP1 and OMP25). Our results have shown that foreign genes were expressed by rSPPV both in permissive (lamb testicles) and non-permissive (bovine kidney, saiga kidney, porcine kidney) cells. Mice immunized with rSPPV(RRΔ)VP1A-(TKΔ)OMP25 elicited specific antibodies to both SPPV and foreign genes VP1 and OMP25. Thus, SPPV NISKHI may be used as a potential safe immunogenic viral vector for the development of polyvalent vaccines.

## 1. Introduction

The use of poxviruses as vectors for foreign gene expression was first shown in 1982 by using the vaccinia virus [1]. The Copenhagen strain of vaccinia virus expressing surface glycoprotein of the rabies virus was the first recombinant strain of poxvirus used in the field for mass vaccination [2]. Since then a great number of vaccinia virus strains were used in the expression of genes of various pathogens [3,4]. The application of wild type vaccinia virus as a vector causes undesirable effects, but their highly attenuated strains are excellent vectors as they are either weakly or not replicating in animal cells [5,6]. Thus far, a number of recombinant vaccinia viruses expressing many genes of animal viruses have been developed [7,8].

Like vaccinia virus, capripoxviruses were also used as vectors to express foreign genes for vaccine development [9,10,11]. The genus *Capripoxvirus* includes three members: lumpy skin disease virus, sheep pox virus (SPPV) and goat pox virus. Despite their high genetic homology, only vaccines made from homologous viruses provide effective protection against infection [12]. In addition, local authority approval is required for the use of live capripoxvirus vaccines in infection-free countries. A vaccine vector based on sheep pox virus will become the basis for the development of multivalent vaccines for small ruminants in countries free from other capripoxvirus infections.

Additionally, with a limited range of susceptible animals, capripoxviruses have great potential for use as non-replicable vaccine vectors [13,14]. Non-replicable vaccine vectors combine the safety of an inactivated vaccine (limited replication ability) and the immunogenicity of a live vaccine (expression of target genes and activation of humoral and cellular immunity), which makes them unique.

Since 1980, a large amount of research on capripoxviruses has been carried out at the Research Institute for Biological Safety Problems SC MES RK. Attenuated sheep pox virus strain NISKHI was generated and used as a vaccine in prophylaxis of sheep pox in Kazakhstan, Russia and other countries of the former Soviet Union [15]. Complete nucleotide sequence of virulent SPPV strain A (GenBank ID: AY077833) and attenuated SPPV strain NISKHI (GenBank ID: AY077834) genomes were reported [16]. Comparative analysis of these two viral genomes revealed the presence of 36 single nucleotide replacements, 15 insertions of 1–29 nucleotides and 20 deletions, which together were found responsible for changes in 17 proteins of SPPV strain NISKHI as compared to strain A. 

Considering the wide range of benefits of using recombinant capripoxviruses as vaccine vectors and our available expertise and tools, in this study our objective was to demonstrate the feasibility of the insertion of at least two foreign genes into the SPPV genome and evaluate their expression in vitro and in vivo.

## 2. Materials and Methods

### 2.1. Virus and Cells

The attenuated SPPV strain NISKHI was provided by the Microbial Collection of the Research Institute for Biological Safety Problems RK ME&S–Science Committee (RIBSP), Gvardeiskiy 080409, Kazakhstan. 

Cell cultures used in this study were provided by the Cellular Biotechnology Laboratory of the RIBSP. Primary lamb testicle (LT) cells were cultured at 37 °C in 5% CO_2_ in semi-synthetic near-wall medium (SNM, RIBSP, RK) supplemented with 10% (*v*/*v*) fetal bovine serum (FBS, Sigma, St. Louis, MO, USA). 

SPPV NISKHI was propagated on LT cells in SNM containing 2% FBS for 7–10 days at 37 °C in 5% CO_2_. The viral titer was determined by plaque assay on LT cells [17]. Plaques were visualized by staining the cells using neutral red at 7–10 days post-infection.

### 2.2. Construction of the Integration Plasmids

**Figure 1:**
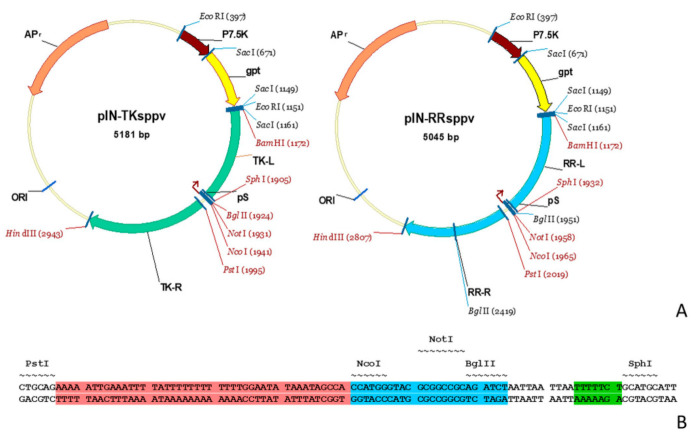
Scheme of DNA plasmid used for the generation of SPPV recombinants. (**A**) pIN-TKsppv and pIN-RRsppv. The vectors consist of the SPPV thymidine kinase (TK) (or ribonucleothide reductase, RR) gene disrupted by synthetic sequence (foreign gene expression cassette), the *Escherichia coli gpt* dominant selectable marker gene under control of the camel pox virus P7.5K early/late promoter, and the ampicillin resistance gene (APr). Arrows indicate the direction of genes transcription. (**B**) Foreign gene expression cassettes consist of synthetic early/late promoter pS (pink), multiple cloning site (blue), stop-codons (white), transcription terminator (green). A multiple cloning site allows for insertion of immuno-protective genes under control of the synthetic early/late promoter pS.

Viral and bacterial DNAs were extracted using the Trizol reagent (Invitrogen, Carlsbad, CA, USA). The targeted sequences were PCR-amplified using suitable primers (Table 1) under the following conditions: 94 °C—5 min, followed by 30 cycles of 94 °C—1 min, 50 °C—1 min, 72 °C—1 min, and a final extension at 72 °C—7 min. Amplification reactions were performed in 50 µL containing 5 µL 10× PCR buffer (containing 25 mM MgCl_2_) (Qiagen, Valencia, CA, USA), 1 µL 10 mM dNTPs (NEB, Ipswich, MA, USA), 0.1 µL template DNA (100 ng/µL), 1 µL of each primer (10 pmol/µL), and 0.25 µL Taq DNA polymerase (1.25 Units, Valencia, CA, Qiagen). All the PCR-amplified DNA fragments were initially cloned in the pGEM-T vector (Promega, Madison, WI, USA) and sequenced. The DNA fragments treated with the corresponding restriction enzymes were inserted successively into the pUC19 plasmid. As a result, the base integration plasmids pIN-TKsppv and pIN-RRsppv were obtained (Figure 1A). The structure of these plasmids was confirmed by restriction analysis and sequencing. The xanthine-guanine-phosphoribosyl transferase gene (*gpt*) was cloned downstream of the P7.5K promoter of camel pox virus and was used as a positive selection marker [18]. Restriction enzyme sites for cloning foreign protein genes were flanked by a synthetic poxvirus promoter on one side and the transcription terminator on the other side (Figure 1B). 

Genes encoding the enhanced green fluorescent protein (EGFP), VP1 protein of Foot-and-mouth disease virus type A (FMDV), and Brucella outer membrane protein 25 (OMP25) were inserted in NcoI and NotI or BglII sites of pIN-TKsppv and pIN-RRsppv. As a result, pIN-TK-EGFP, pIN-RR-EGFP, pIN-TK-OMP25 and pIN-RR-VP1 constructions were obtained. The EGFP gene was synthesized according to the sequence GenBank ID: GU062789. The VP1 gene was RT-PCR-amplified from viral RNA of FMDV type A, isolate Kirgizia/11/2011 (GenBank ID: JQ765587). The OMP25 gene was PCR-amplified from genome DNA of *Brucella abortus*, S19 strain (GenBank ID: CP000887).

### 2.3. Generation and Identification of Recombinant Viruses

Monolayer of LT cells were infected with SPPV NISKHI at 0.1 pfu/cell for 4 h and then transfected with one of the integration plasmids using the Lipofectamine 2000 (Invitrogen, Carlsbad, CA, USA) by following the manufacturer’s protocol. Five days after transfection once cytopathic effect (CPE) reached 80%, the cells were lysed by three cycles of freeze–thaw. For enrichment of the transfection pool with recombinant SPPV, 2–3 successive passages in the selective medium (SNM containing 2% FBS, 2.5 µg/mL mycophenolic acid, 25 µg/mL xanthine and 1.5 µg/mL hypoxanthine) were performed. The resulting recombinant viruses were plaque-purified using an agarose overlay without selective pressure. For plaque, cell lysates were treated with trypsin-EDTA (Gibco, UK) at 37 °C for 30 min to break up clumps. 

Recombinant viruses were confirmed by PCR. For each locus, primers flanking the foreign gene insertion site were designed and used to differentiate recombinant viruses from wild-type viruses (Table 2). In addition, primers binding to the foreign gene were used as internal controls. The PCR protocol mentioned above was used for TK recombinant viruses. To identify RR recombinant viruses, PCR was performed in 50 μL containing 5 μL 10× PCR buffer (containing 25 mM MgCl_2_) (Qiagen, Valencia, CA, USA), 4 μL 25 mM MgCl_2_ (Qiagen, Valencia, CA, USA), 1 μL 10 mM dNTPs (NEB, Ipswich, MA, USA), 0.1 μL template DNA (100 ng/μL), 1 μL of each primer (10 pmol/μL), and 0.25 μL Taq DNA polymerase (1.25 Units, Valencia, CA, USA, Qiagen) and sterile distilled water to the final reaction volume. Template DNA was denatured for 30 s at 94 °C, primer annealing was carried out at 45 °C for 30 s, and strand extension was at 60 °C for 3 min (repeated through 30 amplification cycles).

### 2.4. Light and Fluorescent Microscopy

Cell culture plates were examined using an AxioScope.A1 microscope (Carl Zeiss Microscopy GmbH, Jena, Germany) with an AxioCam ICm1 digital camera and ZEN2012, version 1.1.1.0, Software for Digital Microscopy; Carl Zeiss Microscopy GmbH Germany, 2012). 

### 2.5. Western Blot Analysis

For immunodetection cell lysates were separated in 12% SDS-PAGE, transferred to a nitrocellulose membrane (Invitrogen, Carlsbad, CA, USA). After protein transfer, the nitrocellulose membrane was briefly incubated for 1 h at room temperature (RT) in blocking buffer (1× PBS, containing 0.1% (*v*/*v*) Tween 20 and 5% non-fat dry milk). Then, the membrane was probed with anti-FMDV type А Guinea pig serum (ARRIAH, Russia) or anti-OMP25 mouse serum (RIBSP, Kazakhstan) using a dilution of 1:5000 for two hours at RT, washed three times with PBS-Tween 20 (1× PBS supplemented with 0.1% (*v*/*v*) Tween 20) and incubated in alkaline phosphatase-conjugated anti-guinea pig or anti-mouse antibody for 1–2 h at RT. After three washes with PBS-Tween 20, the membrane was developed by utilizing the BCIP/NBT Phosphatase Substrate Kit (Invitrogen, Frederick, MD, USA), according to the manufacturer’s instructions.

### 2.6. Mouse Immunization

In total, 30 female six-week-old BALB/c mice (weight 25 g) were separated into three groups. Groups 1 and 2 were immunized subcutaneous with 10^5^ pfu rSPPV(RR-)VP1-(TK-)OMP25 and SPPV NISKHI in 100 μL of PBS, respectively. The control group of mice was injected with 100 μL of PBS. All groups were boosted with an equivalent dose at day post-inoculation (DPI) 14. Serum samples were collected at DPI0 and DPI35 and tested by enzyme-linked immunosorbent assay (ELISA).

All experimental procedures and animal care were performed in compliance with institutional animal care regulations. The experiment protocol was approved by the Committee on the Ethics of Animal Experiments of the Research Institute for Biological Safety Problems SC MES RK (Permit Number 06/2017, approved 20 August 2017).

### 2.7. ELISA

The ability of recombinant viruses to induce production specific antibodies were determined by ELISA. Ninety-six-well plates were coated with purified recombinant proteins SPPV095, OMP25 or VP1A (200 ng per well) in 0.1 M carbonate/bicarbonate buffer (pH 9.6) overnight at 4 °С. The plates were then washed three times with TBST buffer (150 mM NaCl, 20 mM Tris-HCl, pH 7.5, 0.05% Tween-20) and blocked with 5% fat-free dry milk in TBST for 1 h at 37 °C. The plates were incubated with duplicates 100-fold dilutions of test sera for 1 h at 37 °C. After washing, alkaline phosphatase conjugated anti-mouse IgG at 1:5000 dilution was added for 1 h at RT. Plates were washed and p-Nitrophenyl Phosphate (pNPP) substrate was added. The optical density was measured after 30 min using dual wavelength reading at 405/630 nm. Samples were considered positive, the optical density of which was at least two-fold greater than in control serum samples. 

Bacterial expressed proteins and mouse antisera were obtained as described [19,20]. Clouse, target genes were cloned into the bacterial expression vector pET26b (containing His6-tag on the C-terminus). Recombinant plasmids were transformed into *Escherichia coli* strain ER2566 (NEB, Ipswich, MA, USA). Bacterial cells were grown in LB-kan (Luria–Bertani broth, containing 50 µg/mL of kanamycin) at 37 °C to OD600 = 0.6–1.0. Gene expression was induced by 1 mM IPTG with subsequent incubation for 2 h at 37 °C. Recombinant proteins were purified by metal-affinity chromatography under denaturing conditions using Ni-NTA-agarose (Novagen, Madison, WI, USA) according to the manufacturer’s protocol. The purified proteins were combined with Montanide Gel-01 (Seppic, Puteaux, France) in a 9:1 ratio. For antibody production, mice were immunized twice at an interval of two weeks. Three weeks after the second immunization, the animals were bled.

### 2.8. Statistic Analysis

Antibody responses are expressed as the mean ± standard deviation of the mean of three independent experiments. The significance of the differences between groups was analyzed using ordinary one-way ANOVA followed by Tukey’s multiple comparisons test. *p* values < 0.05 were considered significant. Statistical analysis of experimental data was performed by using Graphpad Prism, version 6.0, Graphpad Software Inc., San Diego, CA, USA.

## 3. Results

### 3.1. Design and Construction of Integration Plasmids for SPPV Genome Recombination

Integration plasmids for poxvirus recombination include the virus genome regions flanking the targeted gene. Genes encoding proteins nonessential for virus replication in cell culture were used as targets. Thymidine kinase gene is the first and most widely used site for insertional mutagenesis of poxviruses [21,22]. In addition, it was shown that the knockout of the ribonucleotide reductase small subunit gene does not affect the replication of capripoxviruses and can be used to insert foreign sequences [14]. Based on analysis of SPPV NISKHI genomic sequence, the SPPV020 (ribonucleotide reductase, RR) and SPPV066 (thymidine kinase, TK) genes were chosen as insertion sites [16]. 

The foreign gene expression cassette contains the synthetic early/late promoter (pS) [23], terminator T5NT, and multiclonal site (MCS) (Figure 1B). MCS was synthesized as two oligonucleotides (Table 1) and cloned into the SphI–PstI restriction sites between the left and right sequences flanking the insertion sites.

Transient dominant selection method (gene *gpt* as a selective marker) was chosen for generation of recombinant viruses [24]. Selective marker gene *gpt* under the control of the P7.5 promoter was inserted into integration plasmids outside the poxvirus sequences (Figure 1A). This approach allows one to obtain recombinant viruses that do not contain a selective marker gene in their genome. Therefore, using one selective marker gene several mutations can be sequentially introduce into different locus of one viral genome.

As a result, two integration plasmids pIN-RRsppv and pIN-TKsppv for recombination of the sheep pox virus genome were obtained (Figure 1A). The EGFP, VP1A or OMP25 genes were inserted into the MCS of pIN-TKsppv and pIN-RRsppv, resulting in pIN-RR-EGFP, pIN-RR-VP1A, pIN-TK-EGFP, and pIN-TK-OMP25.

### 3.2. Generation of Recombinant Viruses Expressing EGFP

To validate the effectiveness of the obtained expression cassettes, recombinant viruses expressing EGFP were first generated. The use of EGFP eliminates the need for chemical substrates and colorimetric analyzes when evaluating an expression cassette, and requires a luminescent microscope.

**Figure 2:**
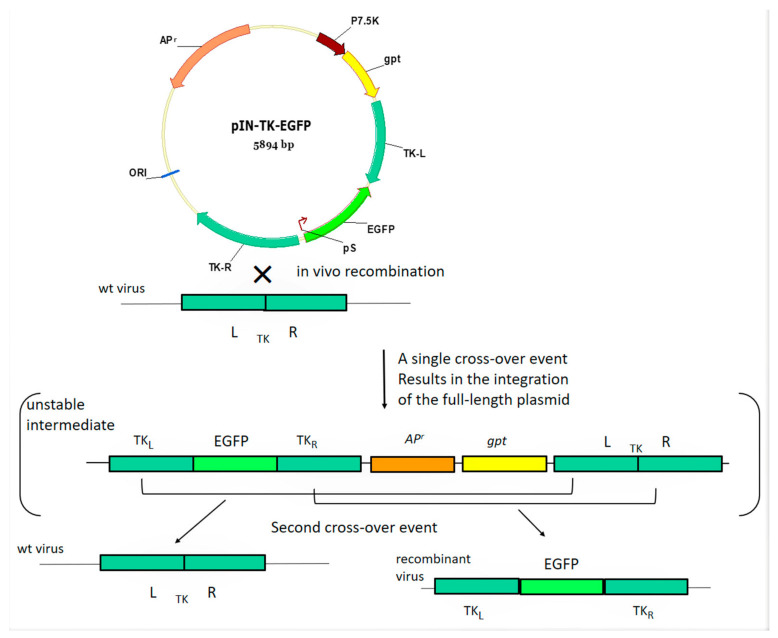
The general scheme for obtaining SPPV with the insertion of the green fluorescent protein gene into the thymidine kinase gene (explanation in the text).

**Figure 3:**
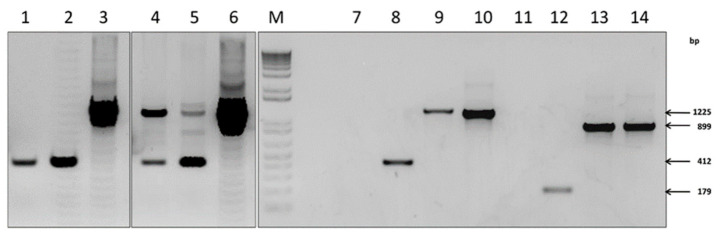
Verification of insertions by PCR. M—1 Kb Plus DNA Ladder (Invitrogen, USA). Lanes 1–10 contain DNA fragments amplified from the TK gene and 11–14 from RR gene; 1, 2—total DNA from LT cells of first selective passage of viruses; 4, 5—total DNA from LT cells of second passage of viruses after selective pressure removal; 7, 11—total DNA of healthy LT cells (control-mock); 8, 12—total DNA from LT cells infected with SPPV NISKhI; 9, 13—total DNA from LT cells infected with the rSPPV(TK-)EGFP and rSPPV(RR-)EGFP, respectively; 3, 6, 10—pIN-TK-EGFP and 14—pIN-RR-EGFP.

SPPV-infected LT cell monolayer was transfected with recombinant plasmid pIN-RR-EGFP or pIN-TK-EGFP under conditions of *gpt*-selection of SPPV recombinants. Single crossing-over leads to integration of plasmid DNA into the viral genome (Figure 2). This genetic construction is unstable and can exist only under selective pressure of mycophenolic acid. Intramolecular recombination of the viral genome at homology regions occurred after removal of the selective conditions, wherein *gpt* gene was cut-out and two types of viruses formed; the recombinant with a targeted gene and the original wild type variant (Figure 2). The recombinant SPPVs with mutant TK or RR genes expressing EGFP were obtained after selection and cloning by plaque method. Plaques formed by recombinant viruses differed from those formed by the parental virus by the presence of green fluorescence. The size and shape of the plaques were similar. The cloning of recombinant viruses was accompanied by PCR analysis (Figure 3). At first, clones containing the foreign gene were selected. Then, these clones were tested for the absence of plasmid DNA. Genetically unstable recombinants containing the plasmid DNA were characterized in PCR as the wild type virus (Figure 3, lanes 1, 2). Two PCR-products corresponding in size to the wild type and recombinant viruses were observed for a mixture of viruses obtained after the removal of selective pressure (Figure 3, lanes 5, 6). Genetically stable recombinants were obtained by cloning the mixture of viruses (Figure 3, lanes 9, 13). PCR fragments of RR and TK genes of the wild type virus were 179 bp and 412 bp long, respectively, while the length of PCR fragments of recombinant virus was 899 bp and 1225 bp.

### 3.3. Effectiveness Reproduction of Recombinant SPPV In Vitro

**Figure 4:**
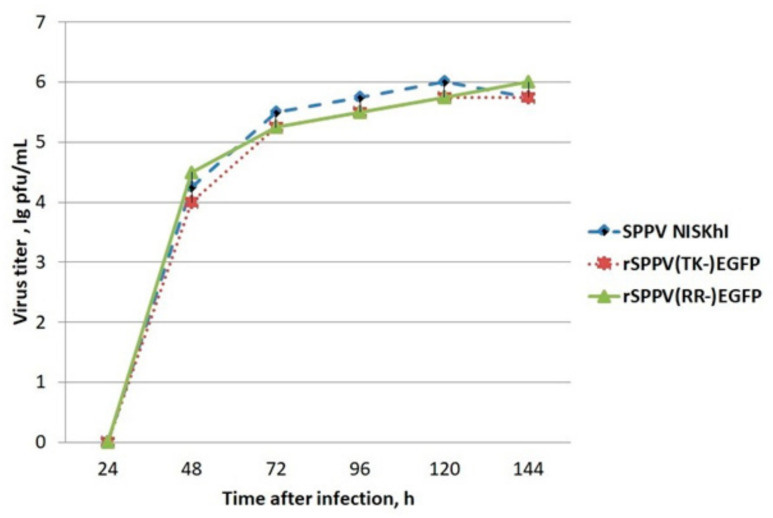
Growth curves for SPPV mutants in LT cells.

After targeted genome recombination it is important to analyze whether the replication of the resulting recombinant virus is retained in cell culture or not. The replication of recombinant sheep pox viruses was performed in LT cells. The SPPV titers were determined by observing the cytopathic effect induced by the virus. Our results showed replication of recombinant viruses rSPPV(TK-)EGFP and rSPPV(RR-)EGFP comparable to the original SPPV NISKHI (Figure 4).

### 3.4. Genetic Stability of Recombinant SPPV

**Figure 5:**
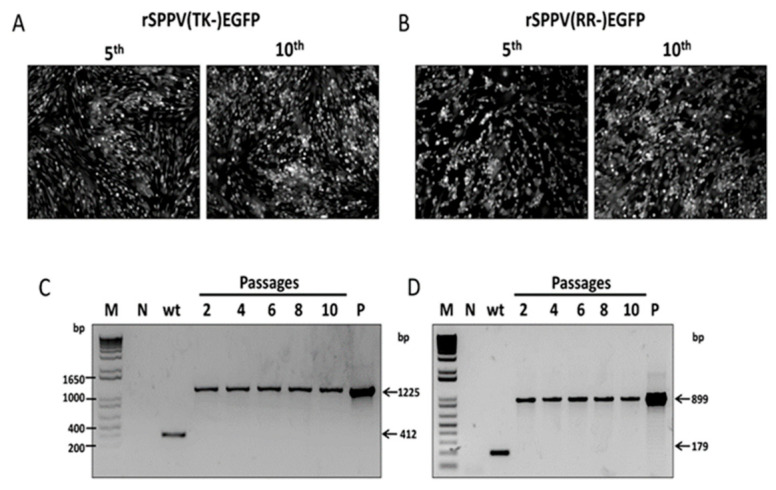
Analysis of genetic stability of the recombinant viruses by fluorescent microscopy (**A**,**B**) and PCR (C, D). (**A**,**B**) Expression of EGFP during passage was detected by fluorescent microscopy (10×). The TK or RR genes were amplified from the DNA of LT cells infected by rSPPV(TK-)EGFP (**C**) or rSPPV(RR-)EGFP (**D**), respectively, as templates after 10 passages. The DNA of LT cells infected with the SPPV NISKhI was used as negative control (wt) and pINT-TK-EGFP and pINT-RR-EGFP were used as positive control (P).

It has been shown that rSPPV(RR-)EGFP and rSPPV(TK-)EGFP recombinants were viable and stably retained the inserted genes even after 10 passages (Figure 5C,D). The presence of targeted gene in the viral vector genome was confirmed by sequencing. Expression of the targeted proteins by rSPPV(RR-)EGFP and rSPPV(TK-)EGFP recombinant viruses during the course of 10 passages were confirmed by luminescent microscopy (Figure 5A,B).

### 3.5. Assessment of EGFP Expression by Recombinant SPPV in Cells of Non-Permissive Hosts

**Figure 6:**
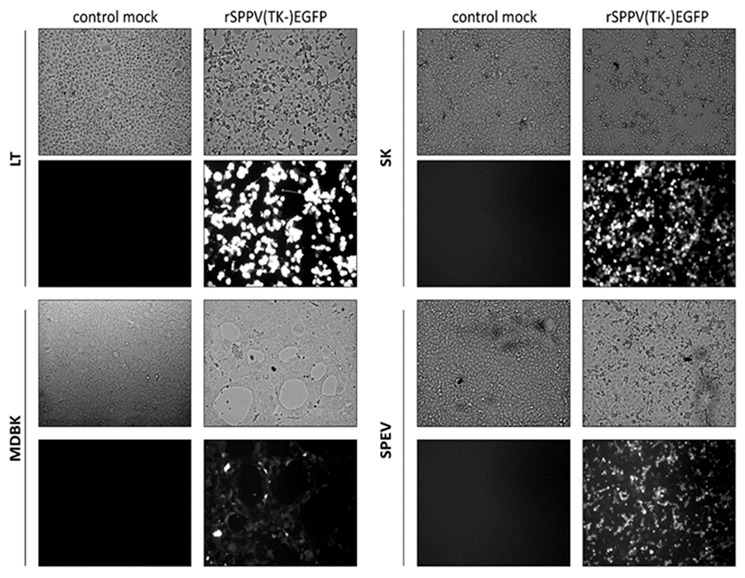
Assessment of EGFP gene expression by fluorescent microscopy (10×). Cytopathic effect of the virus and expression analysis of EGFP in cells of permissive (LT) and non-permissive (SK, MDBK and SPEV) hosts.

*Capripoxvirus* has a limited host range. It can be used to develop both replicating and non-replicating vector vaccines. The ability to express inserted genes in the cells of non-permissive animals will allow the SPPV to be used to develop vaccines not only for small ruminants, but also for other animal species. To demonstrate the expression of foreign genes in cells of non-permissive hosts in vitro, the cell lines MDBK (Madin-Darby bovine kidney line), SK (saiga kidney cell line), SPEV (porcine embryo kidney line) were infected with the recombinant rSPPV(TK-)EGFP virus at a dose of 0.1 pfu/cell. The expression of EGFP gene was detected by fluorescent microscopy. It has been determined that the target protein is expressed both in cells of permissive (Figure 6, LT) and non-permissive (Figure 6, SK, MDBK, SPEV) hosts. However, the amount of protein expressed in cells of non-permissive hosts was much lower than cells of permissive hosts infected using the same dose of recombinant virus. The same data were obtained when evaluating expression of EGFP by rSPPV(RR-)EGFP.

### 3.6. Generation of Recombinant Virus Expressing VP1A and OMP25 Proteins

**Figure 7:**
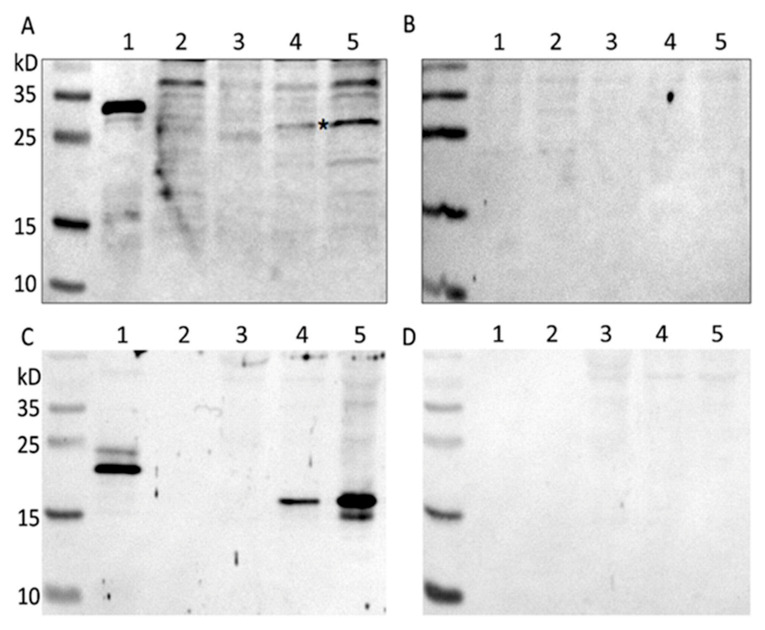
Western blot analysis of proteins expressed by rSPPV(RR-)VP1A-(TK-)OMP25 in LT cells. Protein gel loaded with 5 µL of purified bacterially expressed protein VP1A (**A**,**B** lane 1) and OMP25 (**C**,**D** lane 1), and 10 µL of lysate of cells: LT not infected (**A**–**D**, lane 2), MDBK not infected (**A**–**D**, lane 3), LT infected with rSPPV(RR-)VP1A-(TK-)OMP25 (**A**–**D**, lane 5), MDBK infected with rSPPV(RR-)VP1A-(TK-)OMP25 (**A**–**D**, lane 4). Anti-FMDV type А (**A**) and normal Guinea pig sera (**B**), anti-OMP25 (**C**) and normal (**D**) mouse sera were used to develop the blots. Spectra Multicolor Broad Range Protein Ladder (Thermo Scientific). Asterisks indicated the VP1A protein (**A**). The molecular weight of the bacterially expressed proteins is greater than that of the proteins expressed by the sheep pox virus due to the presence of His6-tag.

Multicomponent vaccines have numerous advantages over individual vaccines, as they reduces the number of vaccinations, dropouts, stress and are easy for follow up and record keeping [25]. Recombinant sheep pox virus expressing protective antigens of several infectious agents can be a good alternative to multicomponent vaccines. The ability of sheep pox virus to simultaneously express both viral and bacterial genes has been evaluated. The procedure was the same as described for the generation and selection of SPPV recombinants expressing EGFP. LT cells infected with SPPV NISKHI were transfected with pIN-TK-OMP25. After selection and isolation, the recombinant SPPV(TK-)OMP25 was obtained. The plaques formed by the recombinant SPPV(RR-)VP1A-(TK-)OMP25 had a size and shape similar to the plaques formed by recombinants with a single insert. After six days of cultivation, the virus activity reached 5.6 ± 0.4 lg pfu/mL. One of the recombinant virus clones was used to insert the VP1A gene into the RR gene using the integration plasmid pIN-RR-VP1A. As a result of selection, the recombinant virus SPPV(RR-)VP1A-(TK-)OMP25 was obtained. The expression of the inserted genes by the recombinant SPPV(RR-)VP1A-(TK-)OMP25 was determined by the Western blot method. The results of these experiments showed that foreign proteins are successfully expressed by recombinant sheep pox virus in both permissive (LT) and non-permissive (MDBK) host cells (Figure 7).

### 3.7. Immunogenicity of Recombinant SPPV Expressing VP1A and OMP25 Proteins

**Figure 8:**
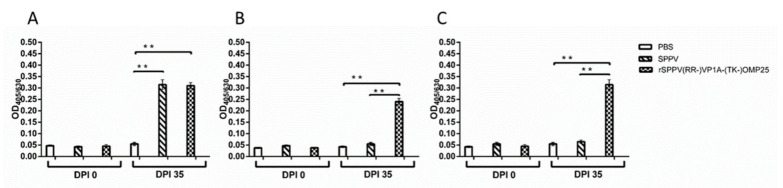
Immune response to rSPPV(RR-)VP1A-(TK-)OMP25 in mice after double subcutaneous immunization. The levels of SPPV095—(**A**), OMP25—(**B**) and VP1A-specific antibodies (**C**) were measured by ELISA. All data are presented as mean ± standard deviation. ** *p* < 0.0001. Statistical analysis was performed using ordinary one-way ANOVA followed by Tukey’s multiple comparisons test. *p* values < 0.05 were considered significant.

The recombinant SPPV(RR-)VP1A-(TK-)OMP25 was grown to high titers in LT cells and its ability to induce an immune response in mice was evaluated. Antibody levels to SPPV095, OMP25 and VP1A in mouse sera were evaluated by ELISA. It was found that the levels of antibodies to the antigen protein SPPV095 did not differ significantly in the groups immunized with SPPV NISKHI and rSPPV(RR-)VP1A-(TK-)OMP25; both were significantly higher than in the control group (Figure 8A). Antibodies to proteins OMP25 and VP1A were found only in the sera of mice immunized with rSPPV(RR-)VP1A-(TK-)OMP25 (Figure 8B,C, respectively).

## 4. Discussion

Liu et al. [21] demonstrate the successful use of two representatives of the genus *Capripoxvirus* (Lumpy skin disease virus and goat pox virus) as vaccine vectors. In this study, the potential of sheep pox virus (the third member of the genus *Capripoxvirus*) to develop multivalent vector vaccines was demonstrated. Recombinant SPPV(RR-)VP1A-(TK-)OMP25 expressed simultaneously two foreign genes both in vitro and in vivo. Mice immunized with rSPPV(RR-)VP1A-(TK-)OMP25 elicited specific antibodies against both SPPV and foreign genes VP1A and OMP25.

For the sequential insertion of two or more mutations (deletions/insertions of genes) into the poxvirus genome by homologous recombination, the selective marker gene must be removed after each recombination. For this purpose, the isolation of recombinant viruses can be carried out under conditions of transient dominant selection [26] or using the Ce/Loxp system [27]. To isolate recombinant SPPVs, the method of transient dominant selection was used. The choice of recombinant selection method determined the structure of the integration plasmids. A selectable marker (*gpt* gene) was inserted into the integration plasmids as a separate expression cassette unrelated to the viral genome sequences (Figure 1A). The ribonucleotide reductase (small subunit) gene and thymidine kinase gene were chosen as sites to inserting foreign genes. DNA sequences flanking the insertion sites were included in the integration plasmid. The sequence encoding the synthetic early/late poxvirus promoter pS, the transcriptional termination signal of the poxvirus early genes T5NT and restrictase sites to cloning foreign gene were located between them (Figure 1B). Since the transcriptional mechanisms of poxviruses do not efficiently recognize eukaryotic promoters, poxvirus promoters must be used to efficiently express recombinant genes in the recipient cell [28]. In this study, synthetic early/late promoter pS and p7.5 promoter of camel pox virus were used to express the target genes. Under the control of the selected promoters, EGFP, OMP25, VP1A and gpt genes were efficiently expressed.

At the first stage, we obtained recombinant viruses SPPV(RR-)EGFP and SPPV(TK-)EGFP expressing the EGFP reporter protein. The insertion of a foreign gene and the elimination of plasmid DNA from the viral genome were confirmed by PCR and sequencing. TK and RR gene knockout did not affect SPPV replication ability. The propagation efficiency in LT cell culture of recombinant SPPV(RR-)EGFP and SPPV(TK-)EGFP was similar to the original NISKHI SPPV (Figure 4). Their good genetic stability also confirmed this fact (Figure 5). The use of EGFP as a model antigen allowed us to evaluate in vitro expression using luminescent microscopy. The EGFP protein was detected at 72 h after inoculating rSPPV(TK-)EGFP into LT, SK, MDBK, and SPEV cells, despite abortive viral infection in MDBK and SPEV cells (Figure 6).

In the next step, we inserted two foreign genes into the sheep pox virus genome. For insertion, the structural protein VP1 of the FMDV and the outer membrane protein 25 of *Brucella* were selected. The FMDV structural protein VP1 is an important antigen and is used for the development of recombinant vaccines [29,30]. *Brucella* OMP25 protein is a protective antigen for brucellosis and is used in combination with other bacterial proteins to develop anti-brucellosis vaccines [31,32]. rSPPV(RR-)VP1A-(TK-)OMP25 was obtained by sequentially inserting the foreign genes VP1A and OMP25 into RR and TK genes, respectively. This recombinant was also genetically stable and efficiently propagated in the LT cell culture.

The ability to express foreign genes and induce an immune response to them is an important indicator of vaccine vectors. In vitro expression of the VP1A and OMP25 genes by rSPPV(RR-)VP1A-(TK-)OMP25 was confirmed by the Western blot (Figure 7). VP1A and OMP25 proteins were recognized by specific sera in both LT and MDBK cells. The expression level was significantly reduced in the cells of non-permissive hosts. A low level of expression of foreign antigens in non-permissive cells is associated with an abortive viral infection.

To evaluate the efficacy of SPPV NISKHI as a recombinant vaccine vector, we investigated the immunogenicity of rSPPV(RR-)VP1A-(TK-)OMP25 in mice. Immunization with rSPPV(RR-)VP1A-(TK-)OMP25 caused specific antibodies against both SPPV and foreign proteins VP1A and OMP25 (Figure 8). The use of a mouse model not only confirmed the in vivo expression of the target genes by the recombinant virus, but also showed the possibility of using the recombinant virus as a non-replicative vector. In the future, it is necessary to select the immunizing dose and the number of vaccine injections to ensure a protective immune response.

## 5. Conclusions

In summary, two integration plasmids with expression cassettes were designed and constructed. Recombinant SPPVs expressing an enhanced green fluorescent protein (EGFP) (rSPPV(RRΔ)EGFP and rSPPV(TKΔ)EGFP), Foot-and-mouth disease virus capsid protein (VP1), and *Brucella* spp. outer membrane protein 25 (OMP25) (rSPPV(RRΔ)VP1A-(TKΔ)OMP25) were generated under transient dominant selection. The insertion of foreign genes into the SPPV020 and SPPV066 open reading frames did not influence the replication of the recombinant viruses in the cells. Our results have shown that foreign genes were expressed by rSPPV both in permissive (lamb testicles) and non-permissive (bovine kidney, saiga kidney, porcine kidney) cells. Mice immunized with rSPPV(RRΔ)VP1A-(TKΔ)OMP25 elicited specific antibodies to both SPPV and foreign genes VP1 and OMP25. 

Safe immunogenic vaccine SPPV, strain NISKHI has potential as a vector for the development of multivalent vaccines. The obtained results provide the basis for future research aimed at improving the vaccine vector to increase its immunogenicity.

## Figures and Tables

**Table 1:** Primers utilized for integration plasmids’ construction.

Primer Name	5′–3′ Sequence ^a^	Amplified Gene	Restriction Site
SPPV066-LF	acggatcctatggatcacagcaagtat	left flanking sequences of SPPV066 insertion site	BamHI
SPPV066-LR	gactgcaggcatgctgcccttcatctatacctataa		PstI-SphI
SPPV066-RF	gactgcagctcgagtctttaaagatattgta	right flanking sequences of SPPV066 insertion site	PstI
SPPV066-RR	gcaagctttcattcatagtcatcctctatatcatt		HindIII
SPPV020-LF	acggatcctatttaaacggtaaattaacttcgt	left flanking sequences of SPPV020 insertion site	BamHI
SPPV020-LR	gactgcaggcatgccagttgagggaatattcttttc		PstI-SphI
SPPV020-RF	gactgcagctcgagcagcaaacgctattaatcgt	right flanking sequences of SPPV020 insertion site	PstI
SPPV020-RR	gcaagcttacgcctttagcataggaactg		HindIII
pS&MCSI-F	gactgcagaaaaattgaaattttattttttttttttggaatataaatagccaccatgggtacgcggccgcagatctaattaattaatttttctgcatgcatt	Synthetic promotor pS and multi-cloning sites	PstI–SphI
pS&MCSI-R	aatgcatgcagaaaaattaattaattagatctgcggccgcgtacccatggtggctatttatattccaaaaaaaaaaaataaaatttcaatttttctgcagtctagagg		
p7.5K-F	ccaggatccgaattcatatactatatagtaataccaa	P7.5 promoter	EcoRI
p7.5K-R	tcggagctcgcgtcactgttctttatgattctactt		SacI
Ec-gpt-F	ccagagctcgccaccatgagcgaaaaataca	*gpt*, xanthine-guanine-phosphoribosyl transferase gene	SacI
Ec-gpt-R	tcagagctcagaaaaattagcgaccggagattggcgggacga		SacI

^a^ Restriction sites are indicated in italics.

**Table 2:** Primers utilized for recombinant viruses’ identification.

Primer Name	Sequence (5’–3’)	Product Size, bp	
		Wild Type	Recombinant Type
PCR-TK-F	aattataggacctatgttttctggc	412	1225 (EGFP)
PCR-TK-R	cagcgtctttataacattccat		1058 (OMP25)
PCR-RR-F	taaacacgcaaaatcacaatg	179	899 (EGFP)
PCR-RR-R	gggctagaaaatggatatcg		799 (VP1A)
EGFP-F	caccatggtgagcaagggcgaggagct	n/a	730
EGFP-R	atgcatgcggccgcttacttgtacagctcgtccatgccgagagtga		
VP1A-F	gctccatgggcgcgcaaaccac	n/a	639
VP1A-R	cgc*g*cggccgtgacatgtcctcctgcatctggtt		
OMP25-F	cacgccatggttgctgccgacgc	n/a	576
OMP25-R	ccaagatctgaacttgatgccgatgccgacgc		

n/a—not applicable.
